# Fibrin glue in ophthalmology

**DOI:** 10.4103/0301-4738.60093

**Published:** 2010

**Authors:** Niranjan Kumar, Khalid Al Sabti

**Affiliations:** 1 Department of Ophthalmology, Al Bahar Ophthalmology Center, Ibn Sina Hospital, Kuwait City, Kuwait; 2 Faculty of Medicine, Kuwait University, Jabriya, Kuwait

Dear Editor,

We read with interest the article by Panda *et al.*,[1] wherein the authors have presented the uses of fibrin glue in ophthalmology in detail. They have mainly discussed its use on the ocular surface as a replacement of sutures.

Fibrin glue has exciting intraoperative applications during vitreoretinal surgeries as well. It has been found to not have any toxic effects on retinal function or structure in a rabbit model.[2] The intraoperative use of Tisseel VH fibrin sealant (Baxter Healthcare Corporation, Westlake Village, CA, USA) has been described recently in the management of optic disc pit-associated macular detachments.[3,4] The technique involves pars plana vitrectomy, removal of posterior hyaloid, fluid-air exchange, drainage of subretinal fluid through optic disc pit, application of the fibrin sealant to the pit and air–C_3_F_8_ gas exchange and postoperative prone positioning. This prevents recurrent macular detachment, which is common in this condition.

Fibrin glue has also been used to stabilize keratoprosthetic devices during vitreoretinal surgeries.[5]
